# GPU Implementation of the Improved CEEMDAN Algorithm for Fast and Efficient EEG Time–Frequency Analysis

**DOI:** 10.3390/s23208654

**Published:** 2023-10-23

**Authors:** Zeyu Wang, Zoltan Juhasz

**Affiliations:** Department of Electrical Engineering and Information Systems, University of Pannonia, 8200 Veszprem, Hungary; zeyu.wang@virt.uni-pannon.hu

**Keywords:** EEG, GPU, Empirical Mode Decomposition, EEMD, CEEMDAN, time–frequency analysis, parallel algorithm, performance

## Abstract

Time–frequency analysis of EEG data is a key step in exploring the internal activities of the human brain. Studying oscillations is an important part of the analysis, as they are thought to provide the underlying mechanism for communication between neural assemblies. Traditional methods of analysis, such as Short-Time FFT and Wavelet Transforms, are not ideal for this task due to the time–frequency uncertainty principle and their reliance on predefined basis functions. Empirical Mode Decomposition and its variants are more suited to this task as they are able to extract the instantaneous frequency and phase information but are too time consuming for practical use. Our aim was to design and develop a massively parallel and performance-optimized GPU implementation of the Improved Complete Ensemble EMD with the Adaptive Noise (CEEMDAN) algorithm that significantly reduces the computational time (from hours to seconds) of such analysis. The resulting GPU program, which is publicly available, was validated against a MATLAB reference implementation and reached over a 260× speedup for actual EEG measurement data, and provided predicted speedups in the range of 3000–8300× for longer measurements when sufficient memory was available. The significance of our research is that this implementation can enable researchers to perform EMD-based EEG analysis routinely, even for high-density EEG measurements. The program is suitable for execution on desktop, cloud, and supercomputer systems and can be the starting point for future large-scale multi-GPU implementations.

## 1. Introduction

Oscillations represent the fundamental mechanism for synchronization and communication among different neural populations in the human brain [1]. Traditionally, the Short-Time Fourier Transform (STFT) has been used to decompose EEG signals into sinusoid components of distinct frequencies [2,3,4]. Unfortunately, the time–frequency uncertainty principle [5] prohibits us from achieving high spectral and temporal resolution at the same time. The Continuous Wavelet Transform using the Morlet wavelet family is an improvement over the STFT [6] as—instead of fixed-sized time windows—it uses frequency-dependent window lengths to extract low-frequency components with higher spectral but lower temporal resolution, whereas high-frequency components have lower frequency but higher temporal resolution [7,8,9]. Unfortunately, both the Fourier and the Morlet Wavelet Transforms require *predefined sinusoid* basis functions of *fixed* frequencies given by analytic formulae as templates, which is not a suitable approach for natural (nonlinear and nonstationary) signals, such as EEG, where the signal shape, amplitude, and frequency can change arbitrarily. 

Empirical Mode Decomposition (EMD) [10] is an alternative frequency decomposition method that decomposes a signal into oscillatory components (Intrinsic Mode Functions, IMFs) without relying on predefined basis functions. Using an iterative method, the IMFs are automatically derived from the signal itself in an adaptive, data-driven way. The extracted IMFs are narrow-band signals carrying instantaneous frequency and phase [11] information as well as amplitude and/or frequency modulation information, which is frequently lost in traditional time–frequency decomposition methods. The original EMD method has some shortcomings, however. The internally used spline interpolation can produce unwanted distortions at the beginning and end of the single (end effects). A more serious problem is known as mode mixing [12], where one IMF contains multiple oscillatory frequencies; i.e., the decomposition into unique frequency components is not accurate. Several new noise-assisted variants, such as Ensemble EMD (EEMD) [13], Complete EEMD with Adaptive Noise (CEEMDAN) [14], and Improved CEEMDAN [15], have been proposed recently to alleviate these problems. All these methods rely on the so-called ensemble approach in which multiple random noise-contaminated copies of the signal are processed to reduce the effect of signal noise. Further, the properties of these methods are described in the Section 2. 

EMD and its variants have been shown to be superior for certain EEG preprocessing tasks and for extracting time–frequency features from EEG data. EMD can be used to remove 50/60 Hz power line noise without notch filtering and distorting the spectrum and phase of the EEG signal [16] and to selectively clean the blink artifact-independent components instead of completely removing them [17,18]. In cognitive experiments, Nguyen et al. [19] showed that EMD—unlike Fourier transform-based analysis—could correctly detect the carrier and modulating signals from flicker-generated steady-state visual-evoked potentials (SSVEPs). Tanaka et al. [20] compared wavelet and bivariate EMD approaches in detecting phase-locking values (PLVs) during a Dynamical Dot Quartet discrimination task and found that EMD was more suitable for detection than the wavelet-based method. Lee et al. [21] and Sweeney-Reed and Nasuto [22] also used EMD for detecting phase synchronization that can be used for brain connectivity network construction or cross-frequency coupling computations. For clinical applications of EMD (BCIs, clinical diagnosis, rehabilitation, seizure detection, sleep staging, anesthesia monitoring, and pain analysis), the reader is referred to the review of Sweeney-Reed et al. [23].

The introduction of multiple noisy copies in EEMD and CEEMDAN results in radical execution time increases. The EEG research community relies heavily on MATLAB as its runtime platform using various EEG toolboxes, e.g., the EEGLAB [24] and Fieldtrip [25], for the analyses with various third-party functions and toolboxes as extensions. The runtime of the EEMD or CEEMDAN methods on a high-density (128 or more channels) and high-sampling rate (*f_s_* over 1 kHz) EEG data file using common MATLAB implementations (Flandrin et al. [26] and EMDLAB by Al-Subari et al. [27]) is measured in hours or days, depending on the sampling frequency and the length of the EEG measurement data. This has a detrimental effect on EEG research, identified as one of the major obstacles to adopting EMD [23], reducing productivity, prohibiting studies with large subject populations, and raising obstacles in research into new data analysis methods. Parallel computing can help in reducing the execution time significantly, especially with graphics processors (GPU) that showed unprecedented performance improvements over the past decade. Unfortunately, achieving highly efficient GPU implementations is a challenging task. Naïve approaches based on common sequential or low-granularity parallel algorithm design techniques often result in disappointing performance, which is an especially serious problem in GPU-accelerated supercomputers that provide only a fraction of their computational performance. 

This paper describes a high-performance GPU implementation of the Improved CEEMDAN algorithm that is designed with performance as a top priority. We minimized expensive data movement and synchronization operations and maximally exploited GPU architecture features that enable efficient execution. The significance of our work is as follows: We achieved over a 260× speedup over MATLAB implementation that reduced the execution time from hours to seconds, and we predicted speedup values in the range of 1000–10,000× for longer measurements. To our knowledge, this is the first GPU implementation of the Improved CEEMDAN algorithm. In addition, we have made the source code of the implementation and sample data files publicly available at https://github.com/EEGLab-Pannon (accessed on 19 October 2023) along with our GPU code for the original EMD and EEMD algorithms as well. We hope that this work will enable the EEG community to routinely use EEMD-based decomposition methods and spectral analyses in its future research. Our implementations also allow users to run large-scale EEG studies on GPU-accelerated cloud systems and supercomputers.

## 2. Materials and Methods

### 2.1. Overview of the Empirical Mode Decomposition Algorithm

Before describing the parallel implementation of the CEEMDAN algorithm, we briefly introduce the basis and the fundamental steps of the original Empirical Mode Decomposition method. The algorithm (described in detail in Algorithm 1) is an iterative method. The input signal is first checked for its extrema points onto which we fit two envelopes (lower envelope on minima, upper envelope on maxima) by cubic spline interpolation. The two envelopes are averaged, and this average envelope is subtracted from the input signal creating a new signal. If this signal satisfies the conditions that make it an IMF, the signal is the new input to the next iteration of the process and the IMF is stored. Once no IMF can be extracted (there is not oscillation in the signal), the process stops. 

Extracting the IMF is performed in the so-called sifting process, which is laid out in details in Lines 5–11 of Algorithm 1. The envelopes are generated for each time sample in every iteration, hence the average and candidate IMF generation is a vector addition and subtraction operation, which can be trivially parallelized. Fitting the envelope requires cubic spline interpolation that, in turn, results in solving a tridiagonal system of equations in each iteration. The innermost while loop of Algorithm 1 controls the sifting process that terminates when a predefined stop criterion has been met [10]. This loop cannot be executed in parallel due to the obvious dependencies between iterations. The outer loop is responsible for controlling the IMF extraction. This process will stop when no further oscillation can be detected in the signal. This loop must also be executed sequentially.
**Algorithm 1**. EMD: Empirical Mode Decomposition [10]Input: x(k)—single-channel time series Output: IMF(k)—N extracted Intrinsic Mode Function time seriesset configuration parametersi = 0**while** (IMF stopping criterion is not met)create a working copy of the input signal: x’(k) = x(k)**while** (sifting stopping criterion is not met)find extrema locations of x’(k)perform cubic spline interpolation on the extrema to obtain the upper and lower envelope of the working copy of the signalcompute the mean of the upper and lower envelopes: m(k) = (upper(k) + lower(k))/2subtract the mean envelope from the working copy: s(k) = x’(k) − m(k)x’(k) = s(k)**end**IMF[i](k) = s(k)x(k) = x(k) − IMF[i] (k)i = i + 1**end**

Several parallel implementations have been developed for the EMD algorithm (For the sake of completeness, we could also include PyEMD in the comparisons, but the PyEMD implementation is fully sequential and significantly slower than libeemd. In addition, Python is less frequently used in the EEG community than MATLAB). The library libeemd [28] is written in the C programming language and provides sequential and OpenMP-based parallel CPU implementations for the EMD, EEMD, and CEEMDAN algorithms. The implementation achieves around a 10× speedup compared to MATLAB ones. The rapid rise of GPU technology in High-Performance Computing gave rise to several parallel GPU-accelerated EMD implementations, too. Waskito et al. reported the first single-precision CUDA EMD implementation for audio signal processing achieving 29× and 29.9× speedups compared to sequential C versions on C1060 and C2050 NVIDIA Tesla cards, respectively [29,30]. Xie et al. created a CUDA EMD version for seismic data processing that achieved a 4× speedup on a GT240 GPU card [31]. Huang et al. [32] reported a 33.7× speedup on a C2050 GPU using overlapped piecewise cubic spline interpolation technique.

### 2.2. Ensemble EMD Algorithms

To solve the EMD mode mixing problem, Wu et al. [13] proposed a noise-assisted signal decomposition method called the Ensemble Empirical Mode Decomposition (EEMD). This algorithm uses multiple copies (called realizations) of the input signal created by adding random Gaussian noise to the signal before the decomposition process. As a result, the distribution of extreme points of the signal will be more uniform in the statistical sense and become less sensitive to intermittent noise. The number of realizations in EEMD is a problem-dependent configuration parameter but, in general, it is in the order of a few hundreds. Compared to EMD, an additional loop is required for the realizations where each iteration starts with replicating the signal by adding to it some random white noise. Then, these signals will be decomposed individually and independently from one another using the original EMD algorithm. Once the IMFs of each noisy copy are extracted, they are averaged to generate the final IMF set. One deficiency of the EEMD method is that it does not produce a *complete* decomposition, meaning that it is not invertible; the exact original signal cannot be rebuilt from the extracted IMFs as some residual noise will appear in the reconstructed signal. A further complication is that different signal plus noise combinations may result in different number of IMFs, making their averaging process problematic. 

Because the Ensemble EMD has a significantly higher computation cost then EMD due to the large number of noise-assisted copies of the original signal, parallelism in this case is mandatory to achieve acceptable execution times. The implementation by Wang et al. was developed for offline spectrum discrimination of hyperspectral remote sensing images and achieved a 60.62× speedup over a sequential C implementation running on an NVIDIA C1060 Tesla GPU card [33]. In a follow-up paper, they compared serial MATLAB, sequential and multicore C, and their CUDA implementation (C1060 GPU) and found that sequential C is 5 times faster than MATLAB, a quad-core C version is 15 times faster, and the CUDA version is 60 times faster than the MATLAB implementation [34]. Chen et al. developed a real-time CUDA EEMD implementation [35] for anesthesia monitoring purposes. They showed that it is possible to achieve a real-time processing speed with a GTX295 GPU card (31.3× speedup, dual GPU card). 

The reviewed GPU implementations have the following characteristics in common: (i) they use early generation, by now outdated GPU processors and early versions of the CUDA programming language; (ii) the achieved speedup values are relatively modest; and (iii) the source code is not publicly available. 

### 2.3. The Improved Complete Ensemble Empirical Mode Decomposition with Adaptive Noise Algorithm

The Improved CEEMDAN algorithm (referred to as ICEEMDAN in the rest of the paper) was developed by Colominas et al. [15]. This algorithm solves the mode-mixing problem, provides an invertible decomposition, and eliminates early noise IMF components from the IMF set. Unlike in EEMD, where the IMFs are extracted for the different signal plus noise realizations independently and averaged at the end, in ICEEMDAN, the extracted IMFs are averaged during the iterative process, and the average is used to compute the input signal for the next iteration. In addition, noise is added to the signal differently, to control the signal-to-noise ratio and match the frequency spectrum of the noise and the new input signals. For this, the EMD algorithm is executed on the white Gaussian noise with zero mean and unit variance to extract noise IMFs, which will be added to the signal as the IMF extraction proceeds. 

The algorithm, described formally, is as follows: Let *E_k_*(·) represent the operator that returns the *k*th IMF (mode) using the EMD algorithm of its input signal. Let *M*(·) be the operator that returns the local mean of the upper and lower envelopes of the signal it is applied to and let $\langle \cdot \rangle$ be the action of averaging across all realizations. With these operators the ICEEMDAN algorithm executes the following steps [15]:

- Calculate by using EMD the local means of K realizations $x^{\left(i\right)}=x+\beta_{0}E_{1}(w^{\left(i\right)})$ to obtain the first residue $r_{1}=\frac{1}{K}\sum_{k=1}^{K}M\left(x+\beta_{0}E_{1}(w^{\left(i\right)})\right)=\langle M(x^{\left(i\right)})\rangle$.
- At the first stage (k = 1), calculate the first mode: $d_{1}=x-r_{1}$.
- Estimate the second residue as the average of local means of the realizations $r_{1}+\beta_{1}E_{2}(w^{\left(i\right)})$ and define the second mode: $d_{2}=r_{1}-r_{2}=r_{1}-\langle M(r_{1}+\beta_{1}E_{2}(w^{\left(i\right)}))\rangle .$
- For k = 3, …, K calculate the kth residue $r_{k}=\langle M(r_{k-1}+\beta_{k-1}E_{k}(w^{\left(i\right)}))\rangle$.
- Compute the kth mode $d_{k}=r_{k-1}-r_{k}$.
- Go to step 4 for next k.

Notice that when creating the signal plus noise realizations, we add $\beta_{k-1}E_{k}(w^{\left(i\right)})$, i.e., the *k*th IMF of the Gaussian white noise to the signal instead of just white noise, such as $\beta_{k-1}w^{\left(i\right)}$. Also, the amplitude $\beta_{k}$ of the noise mode changes from one iteration to the next as given by $\beta_{k}=\varepsilon_{0}std(r_{k})$, where std() is the standard deviation of the signal.

**Figure 1:**
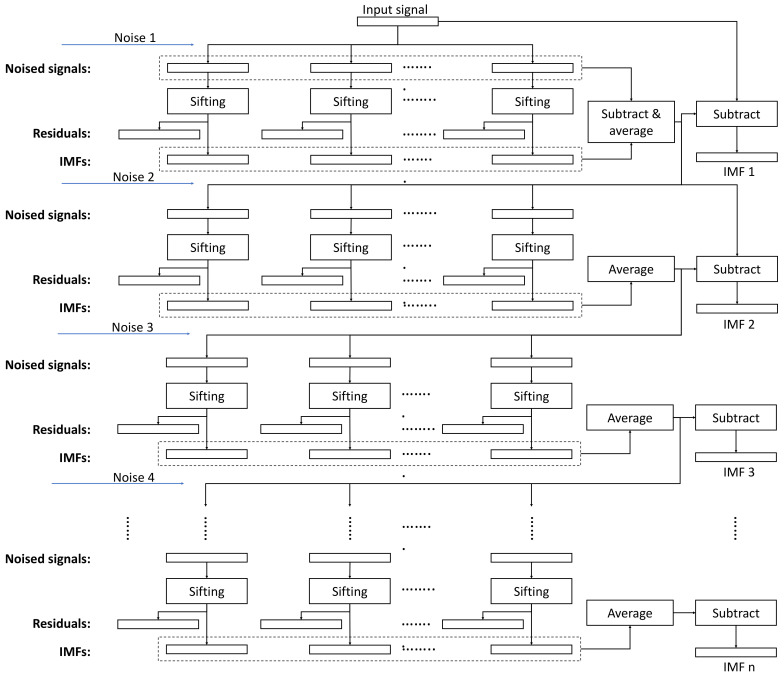
The execution flowchart of the ICEEMDAN algorithm. ‘Sifting’ is the basic processing step of the implementation, and the noise inputs are the decomposition results (IMFs) from the Gaussian noise realizations.

**Figure 2:**
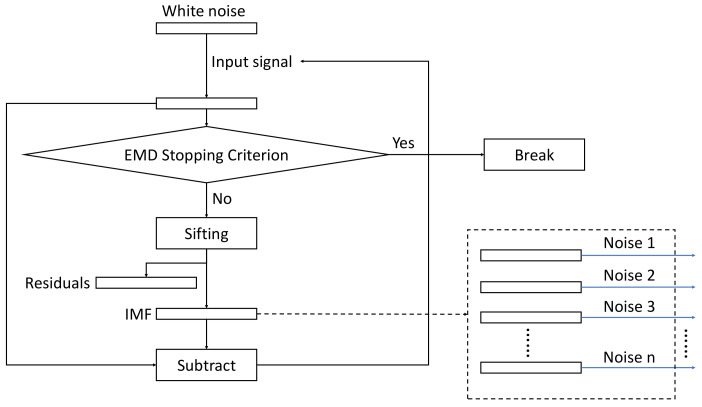
The flowchart of the EMD decomposition of the Gaussian noise. The resulting noise IMFs, Noise 1—Noise n are used as added noise in the algorithm as shown in Figure 1.

The overall structure of the algorithm is depicted in Figure 1, whereas the flowchart of the sifting process for generating the noise IMFs is shown in Figure 2.

### 2.4. Parallel Design

As shown in Figure 1, the ICEEMDAN algorithm has more internal dependencies than EEMD. EEMD for a multichannel EEG dataset can be trivially parallel as the following pseudo-code illustrates: All channels and all realizations can be processed simultaneously as they are independent entities, and only a final reduction step is required to average the IMFs across the realizations for each channel. The degree of parallelism in the double-nested loop is in the order of 10^4^ (Algorithm 2).
**Algorithm 2.** Parallel EEMD Algorithm**for** all channels **perform in parallel****for** all realizations of the current channel **perform in parallel**  *compute IMFs***end for***average IMFs across realizations* **in parallel**
**end for**

On traditional CPU-based parallel systems, this provides enough parallelism so that the IMF computation (EMD) is sufficient to be executed in a serial manner. On GPUs, however, where the number of cores is very close to 10^4^ and the number of parallel threads must be at least two orders of magnitude higher than the core count, the IMF computation should be performed in parallel, too.

The ICEEMDAN algorithm requires synchronization among realizations after each IMF extraction to compute the IMF means to add new random noise and to calculate the input signal for the next iteration. Hence, the high-level structure of the parallel algorithm changes to the following one (Algorithm 3):
**Algorithm 3.** Parallel ICEEMDAN Algorithm**for** all channels **perform in parallel**$\mathrm{generate}\;\mathrm{noise}\;\mathrm{signal}\;w^{\left(i\right)}$$\;\mathrm{and}\;\mathrm{its}\;\mathrm{IMFs}\;E_{k}(w^{\left(i\right)})$ by EMD**for** all realizations **perform in parallel**   $\mathrm{add}\;\mathrm{noise}\;\mathrm{IMF}\;E_{k}(w^{\left(i\right)})$ to signal *x* to obtain the current realization   $\mathrm{compute}\;\mathrm{the}\;\mathrm{local}\;\mathrm{means}\;M(x^{\left(i\right)})$**end for**$\mathrm{residue}\;r_{1}$$\;\mathrm{by}\;\mathrm{averaging}\;M(x^{\left(i\right)})$ across realizations **in parallel**$\mathrm{compute}\;\mathrm{first}\;\mathrm{mode}\;d_{1}$$\;\mathrm{as}\;d_{1}=x-r_{1}$**while** no more IMFs can be extracted **perform** {for *k* = 2 and up}   **for** all realizations **perform in parallel**    $\mathrm{compute}\;\mathrm{the}\;\mathrm{local}\;\mathrm{means}\;M(x^{\left(i\right)})$**end for**   $\mathrm{residue}\;r_{k}$$\;\mathrm{by}\;\mathrm{averaging}\;M(x^{\left(i\right)})$ across realizations **in parallel**   $\mathrm{compute}\;\mathrm{the}\;\mathrm{mode}\;d_{k}$$\;\mathrm{as}\;d_{k}=r_{k-1}-r_{k}$**end while****end for**

We assume the reader is familiar with GPU architectures and programming, and especially with the CUDA programming model. Here, we summarize the terminology we use in the rest of the paper. We kindly refer those with no experience in GPU programming to the CUDA programming literature [36,37,38]. The kernel is a function executing on the GPU device using multiple threads in a single-instruction-multiple-stream fashion. Each thread has a unique index in order to map a thread to a data element in memory. Kernels are launched on the host (CPU program) to be executed on the GPU with threads organized into blocks, and blocks into grids. One-, two- and three-dimensional indexing can be used to map threads onto 1D, 2D, and 3D data structures. NVIDIA GPUs contain a large number of compute (integer, FP32, FP64, and tensor) cores; the internal thread schedulers will assign instructions from threads to different cores based on the operand type for parallel execution. 

### 2.5. The Parallel Sifting Process

The core step of the ICEEMDAN algorithm is the parallel GPU implementation of the sifting algorithm that extracts one mode (IMF) from the input signal. This is used in extracting both the noise and signal IMFs. The main steps of the sifting process are: *extrema detection*, *envelope generation* with cubic spline interpolation, *local mean computation*, and signal *residue calculation*. These steps are implemented by custom kernel functions and in some cases using highly optimized CUDA library functions. The functions used in our sifting process implementation are listed in Table 1.

Each of these steps is executed in a massively parallel fashion. In the extrema detection step, one thread is launched for each signal/noise sample that compares the sample with the left and right neighbors to detect minima and maxima values and their locations. The extrema are used in the next step to generate the upper and lower envelopes of the signal. This is performed by cubic spline interpolation based on the extrema values, which requires the solution of many tridiagonal systems of equations. The Parallel Cyclic Reduction solver implementation /cusparseSgtsv2_nopivot()/ provided in the cuSPARSE library is used for this step. The solver provides us with a set of spline coefficients that we compute in function spline_coefficient(). These are input to the interpolation kernel interpolate() that returns the interpolated values of the upper and lower envelops. The means of these two envelopes are calculated in parallel in function averageUpperLower() as sample wise means. 

Once the sifting process completes, we obtain a new first mode for each realization. These modes are subtracted from their corresponding realizations, and the results are averaged across the realizations to produce the new residue $r_{k+1}$. These steps are executed by kernel functions produceResidue() and averageUpdateSignal(). 

### 2.6. Data Structures and Initialization

The ICEEMDAN algorithm is executed for *C* signal channels, each containing *N* samples. During the decomposition of the signal, we used *I* signal plus noise realizations and extracted up to *K* modes (IMFs). In the following, we describe the most important data structures and their layouts for processing a single channel. The complete list of data structures and their sizes and functions in which they are used are listed in Table S1 in the Supplementary Materials.

The input signal *x* is stored in an *N* element FP32 vector. The *K* modes of the Gaussian noise realizations $w^{\left(i\right)}$ are extracted at the beginning of the program and stored in a *K* × *I* × *N* element three-dimensional FP32 data matrix. Modes from this matrix will be added to the input signal during the iterative process of IMF extraction. Before executing the sifting process, the noisy realizations are generated and stored in an *I* × *N* FP32 matrix. The output of the sifting step is *I* residue signals and *I* modes, both stored in *I* × *N* FP32 matrices. To minimize memory usage, the data structures of the input signal, the residues and IMFs of the realizations are reused and overwritten in each iteration of the IMF extraction loop. As the final result, the extracted averaged IMFs are stored in a *K* × *N* FP32 matrix. 

Only the original input signal and the final IMFs are stored in the host (CPU) memory. All other data structures are allocated to the global GPU memory. The input signal is copied to the GPU memory before the algorithm starts, and the final IMFs are copied back to the host at the end. There are no host–device memory copy operations during the execution of the GPU code. The zero mean and unit variance noise is generated by using the curandGenerateNormal() function in the cuRAND CUDA library.

## 3. Results

In this section, we demonstrate the numerical accuracy, the achieved speedup, and the implementation efficiency of our ICEEMDAN implementation. First, we provide details of the hardware used during the validation and performance measurements. This is followed by numerical validation results and computational performance measures. Finally, we discuss the various performance optimization steps we used to improve execution efficiency and device utilization. 

### 3.1. Test Hardware

Tests and measurements were conducted on three NVIDIA GPUs including gaming and compute-only cards. Each GPU represented different GPU architecture families. Details of the GPUs used in our study are provided in Table 2. Specifically, Titan Xp (Pascal) and RTX 3070 (Ampere) gaming cards were used during development and testing, and a Tesla V100 (Volta) accelerator card was used for additional performance measurements. Because these GPUs have different internal architecture, CUDA core counts, and theoretical peak performance, they enabled us to explore performance differences attributable to varying hardware parameters. For CPU tests, we used an Intel i7-9700K 8-core CPU-based computer with a Windows 10 operating system and MATLAB 2019a. 

### 3.2. Numerical Validation

The numerical correctness of our implementation was validated with a synthetic signal [15] and a real EEG dataset provided as a sample data file in the EEGLAB [24] software distribution. Our GPU implementation was compared with a MATLAB implementation considered as the golden standard [26]. To quantify the accuracy of the decomposition results obtained with different implementations, we introduced the Similarity Index metric ρ given as

(1) $$\rho_{i}(x_{i}\left(t\right),y_{i}(t))=\frac{cov(x_{i}\left(t\right),y_{i}(t))}{\sqrt{var(x_{i}(t))}\sqrt{var(y_{i}(t))}}$$

where cov() represents the covariance of the two input IMF signals $x_{i}\left(t\right)$ and $y_{i}(t)$ produced by the GPU and MATLAB implementations, respectively and var() represents the variance of the input signal. The index ρ varies between 0 and 1, ρ=1 representing that $x_{i}\left(t\right)$ and $y_{i}(t)$ are identical. 

The synthetic signal contains two frequency components and features intermittent noise. The length of the entire signal is 1000 samples, and one component $s_{1}$ is a sinusoid signal with nonzero values from sample 500 to 750 with a frequency of 255 Hz. The other component $s_{2}$ is also a sinusoidal signal but spans the entire signal duration, from sample 0 to 1000 with a frequency of 65 Hz. The composite signal $s=s_{1}+s_{2}$, is expressed as follows:

(2) $$s_{1}=\left\{\begin{array}{cc} 0 & if\;1\leq n\leq 500\\ \sin\left(2\pi 0.255\left(n-501\right)\right) & if\;501\leq n\leq 750\\ 0 & if\;751\leq n\leq 1000 \end{array}\right.\;s_{2}=sin(2\pi 0.065(n-1))$$

**Figure 3:**
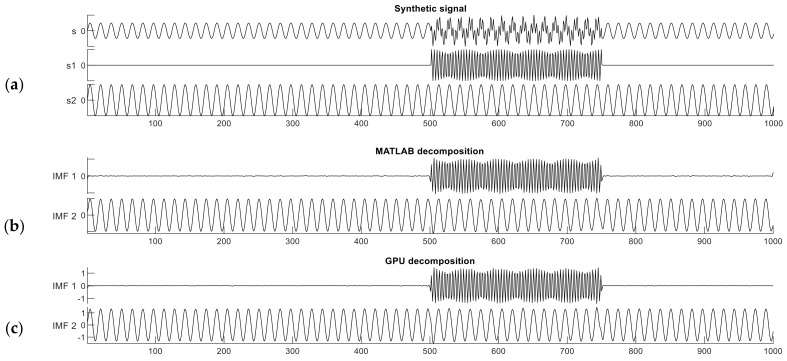
The synthetic dual-frequency signal (**a**) and the decomposition results from the MATLAB (**b**) and CUDA (**c**) implementations.

The synthetic signal *s* and its two constituent components $s_{1}$ and $s_{2}$ are shown in Figure 3a. The interval from sample 501 to 750 in signal s is a period of intermittent noise, which makes the signal well suited for testing mode mixing. 

Figure 3b,c show the decomposition results of this dual-frequency synthetic signal performed with the reference MATLAB and our CUDA implementations. The Similarity Index in this case was computed between one constituent component of signal *s* (the ground truth) and the IMF produced by either implementation; i.e., we measured how accurately the IMF reproduced the original components of the raw synthetic signal. The MATLAB implementation gives similarity index values of $\rho_{s1}^{MATLAB}=99.63\%$ and $\rho_{s2}^{MATLAB}=99.95\%$. The CUDA implementation produced nearly identical results as the MATLAB one, $\rho_{s1}^{CUDA}=99.62\%$ and $\rho_{s2}^{CUDA}=99.91\%$. 

**Figure 4:**
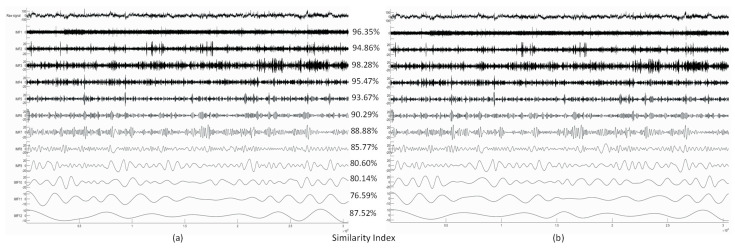
The decomposition results (only IMFs 1–12 are shown, top-down) of Channel 4 from the EEGLAB sample dataset produced by the MATLAB (**a**) and CUDA (**b**) implementations with the corresponding Similarity Index values computed from the corresponding MATLAB and CUDA IMFs.

Next, we show the decomposition results obtained from a real EEG dataset. The selected signal is Channel 4 of the sample data file “eeglab_data.set” distributed with the EEGLAB Toolbox containing 30,504 data samples (sampling frequency is 512 Hz, signal length: 1 min). Figure 4 shows the extracted IMFs and the resulting Similarity Index values. Because we did not have the ground truth in this case, or in the case of any real EEG measurements, the Similarity Index was computed from the MATLAB and CUDA implementation results, treating the MATLAB result as the ‘ground truth’. 

Higher-frequency IMFs show very good agreement of the two implementations. Lower-frequency IMFs show a somewhat reduced level of similarity, which is likely to be caused by the different random number generator in the two implementations and different boundary conditions during extrema detection and spline interpolation. 

### 3.3. Computational Performance and Optimization 

**Figure 5:**
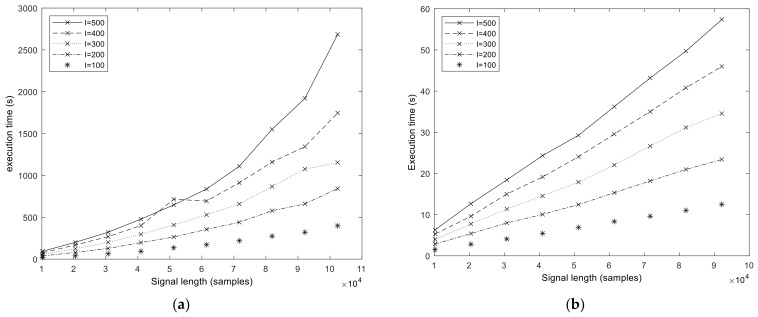
Execution time of the MATLAB (**a**) and libeemd (**b**) versions of the Improved CEEMDAN algorithm in function of signal length *N* and varying number of realizations *I*. The number of sifting operations is fixed, *S* = 10.

We start the performance results section by showing the execution times of the baseline MATLAB and libeemd Improved CEEMDAN implementations (Figure 5). Three input parameters (signal length *N*, number of iterations in the sifting process *S*, and number of realizations *I*) were varied during the tests. It should be noted that because the implementation provided by libeemd uses a completely different iteration stop criterion, we used a fixed number of iterations for a fair comparison. For the sample size *N* = 102,401 that represents 50 s of data at *f_s_* = 2048 Hz or 6.6 min at *f_s_* = 256 Hz, the MATLAB execution time varied between 6 and 53 min depending on the number of realizations (*I* = 100, 200, …, 500) and sifting iterations (*S* = 10, 20, 50). Execution times from the libeemd implementations for the same input parameters varied between 10 s and 4 min.

**Figure 6:**
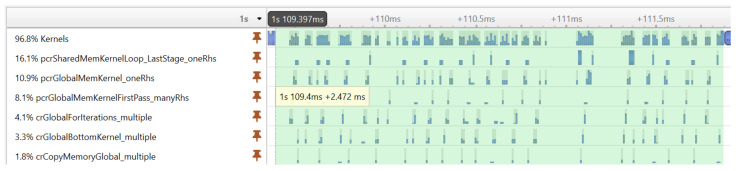
The execution timeline of the kernel functions used by the tridiagonal solver.

Before showing the execution time of our final GPU implementation, we illustrate an important performance optimization strategy. The execution profiling of the first implementation of our algorithm revealed that the cuSPARSE tridiagonal solver executes many small kernels, which—due to the large number of signal realizations—results in a significant performance overhead. Figure 6 shows the execution timeline of the tridiagonal solver on many realizations. It can be seen that the GPU is not fully utilized during the execution of kernels; there are idle time gaps between the kernels.

**Figure 7:**
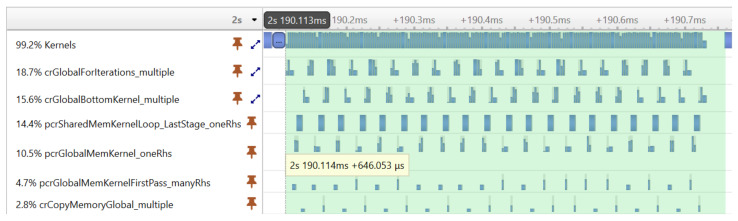
The execution timeline of the kernel functions of the tridiagonal solver using the CUDA graph optimization.

Fortunately, the CUDA programming model provides an elegant solution to the problem of launching many small kernels—the CUDA Graph execution model. With CUDA graphs, one can create a Directed Acyclic Graph from a set of kernels, and later the complete graph can be launched with a single launch call. Graphs can be created programmatically or captured at runtime during program execution. In our version, the latter approach was used. The first execution of the graph was performed by launching the kernels individually to capture the graph. From the second execution, only the captured graph was launched. Figure 7 shows the result of the optimization achieved with CUDA graphs. The same tridiagonal solver was executed as before, but the kernels were now executed much more compactly, without large gaps reducing the execution time from 2.472 ms to 0.646 ms.

**Figure 8:**
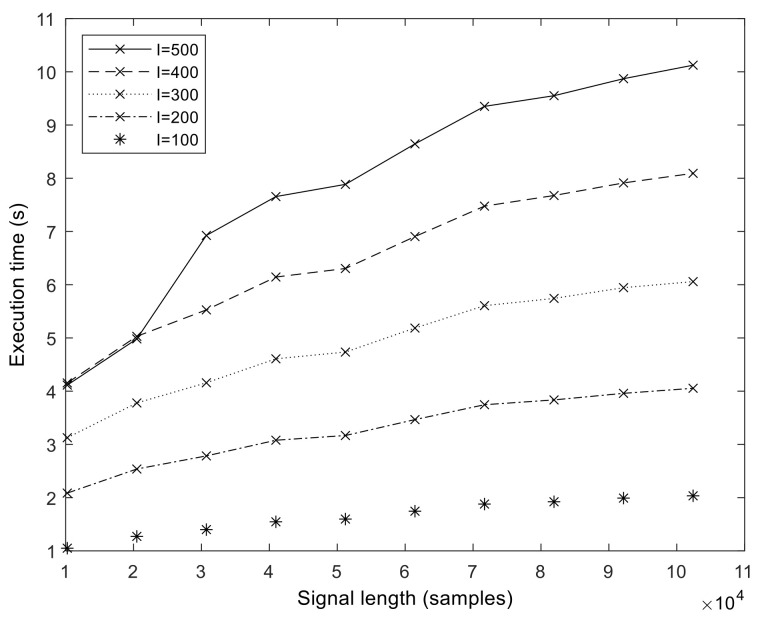
Execution time of the GPU ICEEMDAN algorithm (measured on a V100 GPU) in function of signal length *N* and varying number of realizations *I*. The number of sifting operations is fixed, *S* = 10.

**Figure 9:**
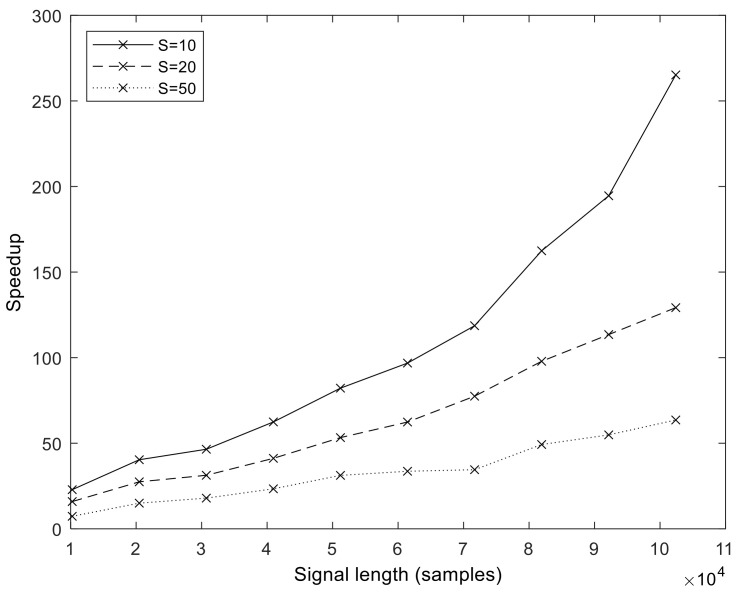
Speedup of the GPU implementation (executed on V100) over the MATLAB version in function of signal length *N* and varying number of sifting iterations *S*. The number of realizations is fixed, *I* = 500.

The execution times of our optimized GPU implementation are shown in Figure 8. The runtime is in the range of 1–10 s for the *N* = 102,401 sample size. We measured the execution time up to *N* = 358,401 samples (representing about 3 min of measurements at *f_s_* = 2048 Hz). From these values, we calculated the speedup values compared to MATLAB, which is shown for a different number of sifting iterations in Figure 9. It is important to note that the speedup increases with sample size and in a superlinear fashion. That is, the more samples we processed, the faster the GPU version became compared to the MATLAB version. The exact speedup values for the full set of realization values are given in Table S2 of the Supplementary Materials. The highest speedup was attained at *N* = 102,401, *S* = 10, and *I* = 500.

**Figure 10:**
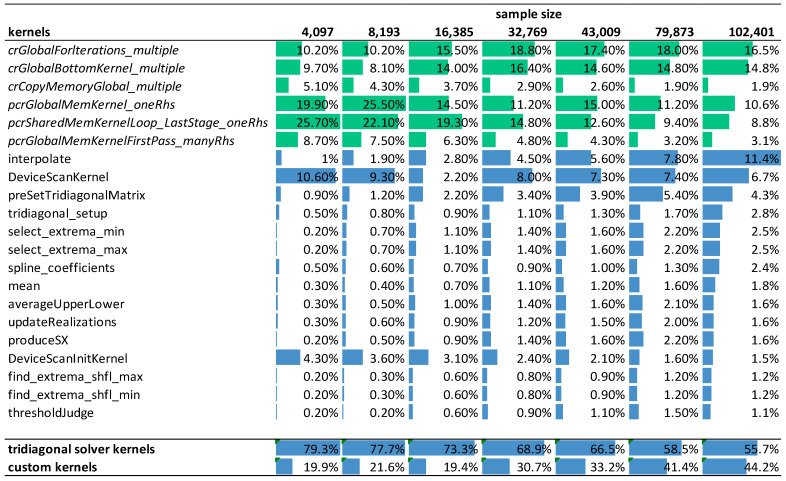
The relative contribution of individual kernels to the overall execution time in function of signal length. Color bars depict the relative weight of the kernels in a given column (green: NVIDIA internal library kernels of the tridiagonal solver, blue: our custom kernels as well as totals).

Next, we show the efficiency of our implementation by analyzing the program execution time and profiling the arithmetic efficiency of kernel functions. Figure 10 shows the relative weight of the GPU kernels during the execution of the ICEEMDAN algorithm in function of signal length on the RTX 3070 mobile card. The number of iterations in the decomposition process was fixed at 10, and the number of realizations was 500. Each column indicates the relative contribution of each kernel to the overall execution time. The green color marks kernels provided by NVIDIA libraries, while the blue color marks kernels we developed ourselves. The last two rows of the table show the total contribution of the CUDA library functions and our customized kernels to the overall execution time. 

The profiling shows a trend that with increasing signal length, our custom kernels accounts for an increasing proportion of the overall execution time with the kernel function interpolate() becoming the dominating factor. The NVIDIA library functions are limiting performance for smaller input data sizes (72% vs. 28%), but as the data size increases, their effect becomes smaller (52% vs. 48%).

**Figure 11:**
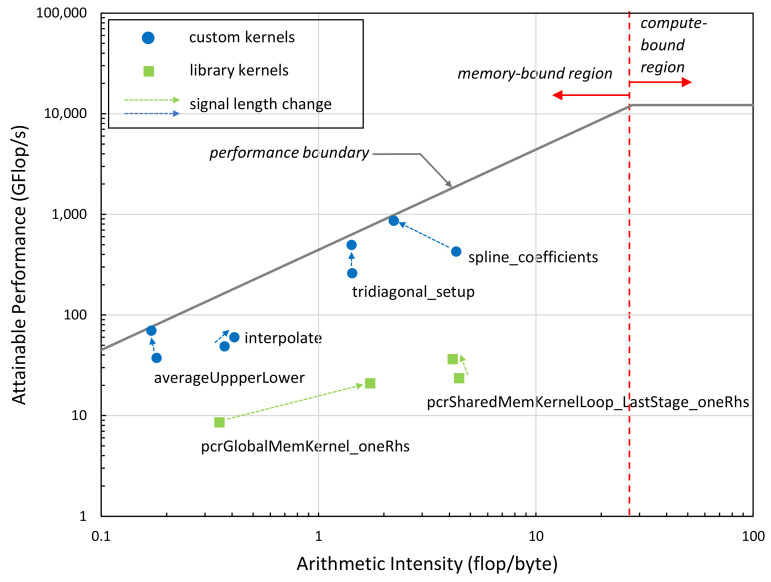
The Roofline performance results of the kernels executed on the RTX 3070 mobile GPU showing the performance positions of the main kernels of the implementation. Arrows indicate performance change as signal length is increased from 4 k to 100 k samples.

We also performed a Roofline performance analysis [39] of the performance-critical kernels of our implementation at two different signal lengths (4 k, 100 k). As can be seen in Figure 11, all kernel functions are memory-bound based on their Arithmetic Intensity (arithmetic operations per number of bytes transferred to/from memory); that is, the performance is limited by the memory bandwidth not by the computational performance of the GPU. The green boxes represent internal kernels of the NVIDIA library, while blue dots represent the kernels we developed. The closer the dots are to the performance boundary, the more efficient the kernels are. Kernels significantly below the line vertically indicate performance problems, typically latency issues. The results indicate that the kernels we developed are closer to the theoretical performance limit (performance attainable at a given arithmetic intensity value) than the NVIDIA kernels. The arrows in the figure indicate the performance change of the kernels when increasing the signal length from 4 k to 100 k. The subsequent change in the kernels’ position in the Roofline diagram suggests that our implementation becomes more efficient as signal size increases.

**Figure 12:**
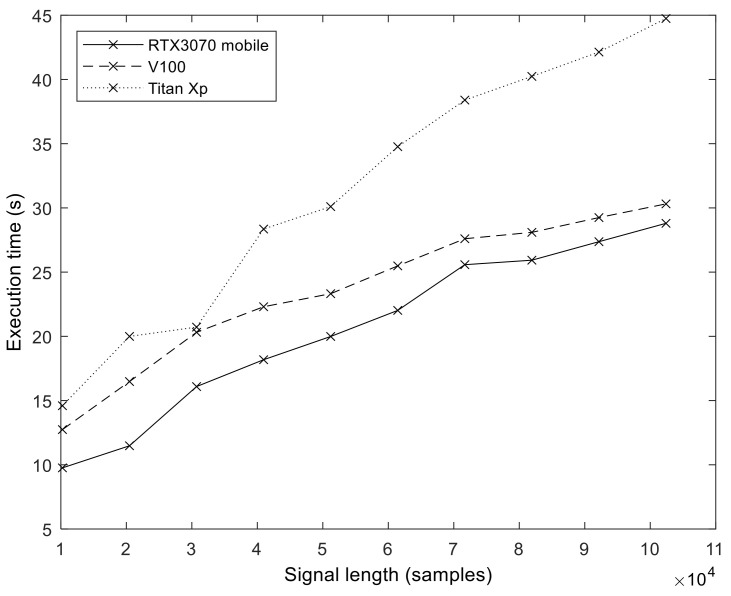
The execution time of the GPU algorithm on three different GPU architectures. The results demonstrate that each newer architecture generation (Pascal -> Volta -> Ampere) provides increased performance for the same program.

In order to explore the effect of GPU hardware architecture on the execution performance of our implementation, we measured and compared the execution times on three different GPUs (see Table 2 for details). Figure 12 shows the execution time values we obtained on the different GPUs (*S* = 100, *I* = 200). The effect of hardware evolution and the introduction of new architectural features is evident. The best results were obtained with an Ampere GPU, followed by Volta. The oldest architecture, Pascal (Titan Xp), produced the longest execution times. 

## 4. Discussion

Oscillations play a key role in understanding how the human brain is coordinated during task execution as oscillations are thought to be the means of communication and information transfer between neural assemblies. Delta (1–4 Hz), theta (4–8 Hz), alpha (8–13 Hz), and beta (13–35 Hz) band oscillations have different but distinct roles in coordinating actions, and their deviation from patterns found in healthy populations may indicate neural degeneration (e.g., Mild Cognitive Decline, stroke, and Parkinson’s disease) and may be used as a means for early diagnostics. 

Detecting oscillation in natural, nonperiodic, and nonstationary signals is a challenge. Traditional methods, such as the Short-Time Fourier Transform or the Continuous Wavelet Transform, can only provide an approximate and crude result, as the exact localization of time and frequency is not possible with these methods. Empirical Mode Decomposition enables us to extract instantaneous frequency and phase information from the EEG signal and hence provides the means for following amplitude, frequency, and phase changes at a very high temporal resolution. The importance of this cannot be underestimated as a new tool that helps uncover the electrophysiological processes of the brain. Phase synchronization information is the basis of computing brain functional connectivity networks that describe the cooperation of different cortical areas in either resting state or during task execution. Traditional methods only allow for the generation of static networks, which is a major disadvantage because the construction and study of dynamic connectivity networks are crucial for understanding how our brain works [40,41,42,43]. Sweeney-Reed [22] and independently our group showed that EMD is suitable for extracting instantaneous phase information and consequently creating dynamic functional networks [44].

Due to mode mixing and mode splitting, variants of EMD have been developed among which the most promising algorithm is the Improved CEEMDAN. Unfortunately, the computational complexity so far has prohibited the widespread use of this method in research. The GPU implementation that we presented in this paper achieved exceptional efficiency and showed over a 260× speedup compared to the MATLAB implementation. The exact speedup values vary with the algorithm’s input parameters (number of sifting iterations, number of realizations, and signal length) but for common settings vary between 65× and 265×. The accuracy of the implementation was validated against the MATLAB version [26]. The results show very high agreement (average Similarity Index > 89.0%) with the MATLAB results. The small difference is due to the different random number generators and seeds used in the two implementations and differences in CPU and GPU floating point arithmetic. 

**Figure 13:**
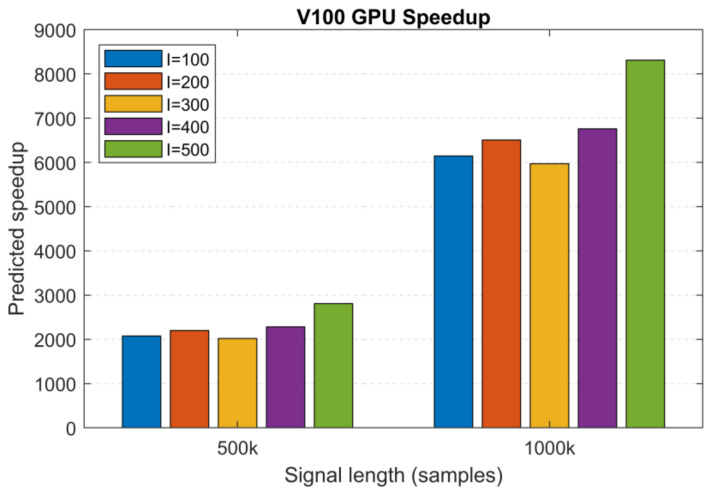
Predicted speedup values for the V100 GPU as a function of signal length based on predicted MATLAB (CPU) execution times (*S* = 10). Increasing the signal length has a significant positive effect on the achievable speedup.

Based on the MATLAB and V100 execution times of two large datasets (*N*_1_ = 500 k and *N*_2_ = 1000 k samples, *I* = 100, *S* = 10, MATLAB: 2 h 42 min and 13 h 13 min and V100: 4.7 s and 7.7 s), we extrapolated the execution times for the remaining 200 to 500 realizations and used these to calculate the predicted speedup values. Figure 13 shows the predicted speedup for *S* = 10 sifting iterations. For the different number of realizations, we obtained >2000× and >6000× speedups for the signal lengths *N*_1_ and *N*_2_, reaching a peak value of 8310× for length *N*_2_ and 500 realizations. Based on these predictions and the effect of architecture on performance, we can safely assume that the most recent GPU generations (Tesla A100, H100) with an increased amount of memory will outperform these results. 

There are known limitations of our implementation. Firstly, our algorithm performs the ICEEMDAN algorithm on a single channel. Multiple channels can be handled by repeated execution of the program for the channels either in a serial fashion using a single GPU or multiple GPUs. If multiple GPUs are available, each GPU may process a single or a set of channels in parallel, in the latter case, one channel after the other. Secondly, the IMFs of the noise signal realizations were generated in advance at the beginning of the program, which requires sufficient GPU memory to hold *K* × *I* × *N* samples. For large sample sizes and a large number of realizations, the GPU memory can easily become a bottleneck. By moving the noise IMF computation into the main signal IMF extraction loop, the required memory space can be reduced and longer signals or more realizations can be processed. In addition, by using the CUDA Unified Memory, the largest data structures can be stored in the host’s memory and loaded to the GPU in an on-demand fashion. However, both of these methods are expected to reduce the achieved performance.

When we compared the GPU execution time to the libeemd times, the difference is not as significant as for the MATLAB implementation. The highest speedup over the libeemd implementation is 6.3×. This is due to the fact that most GPU kernels are memory-bound and the memory bandwidth severely limits the attainable performance. As seen in Figure 11, most kernels achieve up to or below 100 GFlop/s performance. Those that perform near 1 TFlop/s represent a small fraction of the full implementation. This indicates that (i) future systems with higher memory subsystems will perform better and (ii) more work is needed to improve the Arithmetic Intensity of the kernels (e.g., by using kernel fusion) to push the performance higher, toward the compute-bound region. 

## 5. Conclusions

This paper describes a massively parallel GPU implementation of the Improved CEEMDAN algorithm. The ICEEMDAN method is a crucial tool for the precise time–frequency analysis of nonstationary EEG signals. It can be used in various stages of EEG processing, from preprocessing through time–frequency to connectivity analysis, and to calculate instantaneous frequency, power, and phase information in a very short amount of time, enabling researchers to uncover the dynamic properties of brain processes underlying perception and task execution. 

Despite some known limitations, to our knowledge, this is the first GPU implementation of the Improved CEEMDAN algorithm. Here, we present evidence of the efficiency of our implementation reaching potentially a four-orders-of-magnitude increase in computing speed over the most frequently used MATLAB implementation. The source code of the implementation is publicly available under the MIT License at the Github page https://github.com/EEGLab-Pannon/CEEMDAN-GPU (accessed on 19 October 2023) of our group. Our implementation allows users and researchers to perform the decomposition of nonstationary natural signals into oscillatory components almost instantly, opening up new opportunities in research and in applications. 

Future work will include the adaptation of our algorithm for supercomputer execution where hundreds to thousands of GPU cards are available. These systems not only would allow many channels to be executed simultaneously but also enable the datasets of multiple subjects to be performed at the same time, reducing the execution time of oscillation analysis of potentially large groups to seconds. 

## Figures and Tables

**Table 1:** The major steps of the sifting process and their corresponding GPU kernel/library function.

Sifting Operation	Kernel/Library Function
Find local signal maxima	select_extrema_max()
Find local signal minima	select_extrema_min()
Solve the tridiagonal system	cusparseSgtsv2_nopivot()
Collect coefficients for interpolation	spline_coefficient()
Cubic spline interpolation	interpolate()
Compute mean envelope	averageUpperLower()
Signal update for next iteration	averageUpdateSignal()

**Table 2:** Architecture parameters of the GPU platforms used for measurements.

	Titan Xp	Tesla V100	RTX 3070 Mobile
Architecture	Pascal	Volta	Ampere
CUDA cores	3840	5120	5120
Clock frequency (GHz)	1.48	1.46	1.62
Memory (GB)	12	16	8
Peak FP32 performance (TFlop/s)	11.36	14.03	16.59
CUDA version	10.2	11.3	11.4

## Data Availability

Data sharing not applicable.

## Supplementary Materials

The following supporting information can be downloaded at https://www.mdpi.com/article/10.3390/s23208654/s1: Table S1: Device variables used in the GPU implementation, their size and the kernels in which they are referenced; Table S2: Speedup values for different test configurations.
