# Supplementary material for: GPU Implementation of the Improved CEEMDAN Algorithm for Fast and Efficient EEG Time–Frequency Analysis
Source: Sensors (Basel). 2023 Oct 23;23(20):8654. doi: 10.3390/s23208654 (PMC10611056; doi:10.3390/s23208654)
Supplement: Supplementary file 1 [file sensors-23-08654-s001.zip › sensors-2641405-supplementary.pdf]

Table S1. Device variables used in our GPU implementation, their size and kernels in which they are referenced

Variable name	Dimensions	Used in kernel function
d_current	SignalLength × NumNoise	findExtremaShfl(), selectExtrema(), updateRealizations()
d_noisedSignal		produceFirstIMF() addNoise()
d_whiteNoise		curandGenerateNormal(), addNoise()
d_whiteNoiseModes	SignalLength × NumNoise x NumIMFs	addNoise(), updateRealizations(), updateSignal()
d_channelMeans	NumNoise	mean(), multiply()
d_channelVariance	NumNoise	variance(), multiply(), addNoise()
d_sparseFlag	SignalLength × NumNoise	findExtremaShfl(), DeviceScanInitKernel(), DeviceScanKernel(), selectExtrema()
d_noisedSignalIndex	SignalLength × NumNoise	findExtremaShfl(),selectExtrema(), interpolate()
d_ScanResult	SignalLength × NumNoise	scanLargeDeviceArray(), scanSmallDeviceArray(), selectExtrema()
d_compactValue d_compactIndex/	SignalLength × NumNoise	selectExtrema(), setBoundary(), tridiagonalSetup(), splineCoefficients(), interpolate()
d_num_extrema_max/ d_num_extrema_min	NumNoise	selectExtrema(), tridiagonalSetup(), splineCoefficients(), interpolate(), averageUppperLower(), siftingCriterion()
d_upperDia d_middleDia d_lowerDia d_right	SignalLength × NumNoise	preSetTridiagonalMatrix(), tridiagonalSetup() cusparseSgtsv2(), splineCoefficients(), interpolate()
d_meanEnvelope		averageUppperLower(), updateRealizations()
d_envelopeVauleMax/ d_envelopeVauleMin		averageUppperLower(), interpolate(), produceSX()
d_residue		produceResidue(), averageUpdateSignal()
d_forNext	SignalLength	averageUpdateSignal(), produceFirstIMF(), addNoise()
d_running	SignalLength	produceFirstIMF()
d_IMFs	SignalLength x NumIMFs	averageUpdateSignal(), produceFirstIMF()

Table S2. Speedup values for different test configurations (V100 vs MATLAB).

Number of Sifting Iterations	Signal Length	Number of realizations				
		500	400	300	200	100
10	10241	22.9	18.4	18.7	18.5	18.1
	20481	40.4	33.7	32.9	31.8	31.0
	30721	46.5	48.7	48.4	46.0	46.2
	40961	62.4	65.2	64.4	64.4	60.3
	51201	82.2	113.5	86.2	83.4	84.7
	61441	96.8	100.5	102.1	102.6	98.6
	71681	118.7	122.0	117.6	117.7	116.8
	81921	162.4	151.1	151.0	150.7	142.8
	92161	194.6	169.8	181.1	166.9	161.3
	102401	265.2	215.7	190.5	207.7	196.1
20	10241	15.9	11.9	11.9	11.9	11.1
	20481	27.5	19.9	19.5	19.9	18.5
	30721	31.3	29.2	28.9	29.1	27.9
	40961	41.2	39.1	36.9	37.3	35.6
	51201	53.3	50.3	49.3	47.8	48.1
	61441	62.4	58.6	57.4	56.6	53.8
	71681	77.5	65.7	66.7	67.4	63.5
	81921	97.9	90.8	81.5	81.3	77.5
	92161	113.5	92.8	92.9	102.7	90.0
	102401	129.2	111.2	124.7	106.8	105.4
50	10241	7.3	6.6	6.4	6.5	7.0
	20481	15.0	11.5	11.1	11.0	12.3
	30721	17.9	17.7	17.8	17.2	16.4
	40961	23.4	22.4	22.9	21.0	22.2
	51201	31.2	30.0	30.7	30.4	28.5
	61441	33.6	32.4	34.7	32.6	31.5
	71681	34.5	37.8	36.4	35.3	37.0
	81921	49.3	46.0	46.1	44.2	43.3
	92161	54.9	53.8	47.4	51.1	49.4
	102401	63.6	66.2	64.6	54.8	53.4
